# A Fasting Insulin–Raising Allele at *IGF1* Locus Is Associated with Circulating Levels of IGF-1 and Insulin Sensitivity

**DOI:** 10.1371/journal.pone.0085483

**Published:** 2013-12-31

**Authors:** Gaia Chiara Mannino, Annalisa Greco, Carlo De Lorenzo, Francesco Andreozzi, Maria A. Marini, Francesco Perticone, Giorgio Sesti

**Affiliations:** 1 Department of Medical and Surgical Sciences, University “Magna Graecia” of Catanzaro, Catanzaro, Italy; 2 Department of Systems Medicine, University of Rome-Tor Vergata, Rome, Italy; University of Siena, Italy

## Abstract

**Background:**

A meta-analysis of genome-wide data reported the discovery of the rs35767 polymorphism near IGF1 with genome-wide significant association with fasting insulin levels. However, it is unclear whether the effects of this polymorphism on fasting insulin are mediated by a reduced insulin sensitivity or impaired insulin clearance. We investigated the effects of the rs35767 polymorphism on circulating IGF-1 levels, insulin sensitivity, and insulin clearance.

**Methodology/Principal Findings:**

Two samples of adult nondiabetic white Europeans were studied. In sample 1 (n=569), IGF-1 levels were lower in GG genotype carriers compared with A allele carriers (190±77 vs. 218±97 ng/ml, respectively; *P*=0.007 after adjusting for age, gender, and BMI). Insulin sensitivity assessed by euglycaemic-hyperinsulinemic clamp was lower in GG genotype carriers compared with A allele carriers (8.9±4.1 vs. 10.1±5.1 mg x Kg^-1^ free fat mass x min^-1^, respectively; *P*=0.03 after adjusting for age, gender, and BMI). The rs35767 polymorphism did not show significant association with insulin clearance. In sample 2 (n=859), IGF-1 levels were lower in GG genotype carriers compared with A allele carriers (155±60 vs. 164±63 ng/ml, respectively; *P*=0.02 after adjusting for age, gender, and BMI). Insulin sensitivity, as estimated by the HOMA index, was lower in GG genotype carriers compared with A allele carriers (2.8±2.2 vs. 2.5±1.3, respectively; *P*=0.03 after adjusting for age, gender, and BMI).

**Conclusion/Significance:**

The rs35767 polymorphism near *IGF1* was associated with circulating IGF-1 levels, and insulin sensitivity with carriers of the GG genotype exhibiting lower IGF-1 concentrations and insulin sensitivity as compared with subjects carrying the A allele.

## Introduction

A large-scale meta-analysis of genome-wide data for continuous diabetes-related traits in nondiabetic participants conducted by the Meta-Analyses of Glucose and Insulin-related traits Consortium (MAGIC) has reported the discovery of two loci (*IGF1* and *GCKR*) with genome-wide significant association with fasting insulin levels [1]. Another more recent meta-analysis of up to 108,557 individuals of European ancestry without diabetes, including individuals newly genotyped using the Metabochip, have identified 17 additional loci with genome-wide significant association with fasting insulin concentration [2]. The confirmed 19 loci associated with fasting insulin levels may influence circulating insulin concentration by distinct physiological processes, including insulin sensitivity, insulin secretion, and insulin clearance. Among them, the rs35767 polymorphism located 1.2 kb upstream of *IGF1* is a biologically plausible fasting insulin–raising gene. Liver-specific IGF-1 knockout mice exhibit insulin-resistance and hyperinsulinemia that are reversed by recombinant insulin-like growth factor-1 (rhIGF-1) treatment [3]. Additionally, there is evidence that low plasma IGF-1 concentrations are associated with reduced insulin-sensitivity [4], metabolic syndrome [5], and predict development of glucose intolerance and type 2 diabetes [6]. Thus, the rs35767 polymorphism near *IGF1* may affect insulin sensitivity, and therefore, fasting insulin levels, a surrogate for insulin resistance [7]. On the other hand, another polymorphism near *IGF1* (rs35749) has been recently associated with insulin clearance [8]. Because there is evidence that insulin clearance is the strongest determinant of the variability in fasting insulin levels independently of insulin resistance, insulin secretion, adiposity, and fasting plasma glucose [9], it is possible that the association between the rs35767 polymorphism and fasting insulin levels is mediated by variation in insulin clearance. To clarify these issues, we sought to investigate the effects of the rs35767 polymorphism on circulating IGF-1 levels, insulin sensitivity, measured by the gold-standard technique, the euglycaemic–hyperinsulinaemic clamp, and insulin clearance in white Europeans.

## Methods

### Study subjects

Two different samples of adult (≥18 years of age) nondiabetic individuals of European ancestry were studied.

Sample 1 comprised 569 non-diabetic offspring of patients with type 2 diabetes from the EUGENE2 project [10] consecutively recruited at the Department of Medical and Surgical Sciences of the University ‘Magna Graecia’ of Catanzaro according to previously reported inclusion criteria [11]. Participants underwent anthropometrical evaluation including measurements of body mass index (BMI), waist circumference, and body composition evaluated by bioelectrical impedance. A 75 g OGTT was performed with 0, 30, 60, 90 and 120 min sampling for plasma glucose. Insulin sensitivity was assessed by euglycemic-hyperinsulinemic clamp study, as previously described [4,11]. Briefly, a priming dose of insulin (Humulin, Eli Lilly & Co., Indianapolis, IN) was administrated during the initial 10 min to acutely raise plasma insulin followed by continuous insulin infusion fixed at 40 mU/m^2^ x min. The blood glucose level was maintained constant during the 2-h clamp study by infusing 20% glucose at varying rates according to blood glucose measurements assessed by a glucose analyzer at 5 minute intervals (mean coefficient of variation of blood glucose was < 5%). Blood samples for plasma insulin assay were drawn at 60, 80, 100, and 120 min during the clamp study. 

In order to get further insights on the role of the rs35767 polymorphism on IGF-1 levels and insulin sensitivity, an additional sample comprising 859 nondiabetic individuals was analysed. This sample 2 includes individuals consecutively recruited at the Department of Systems Medicine of the University of Rome-Tor Vergata and at the Department of Medical and Surgical Sciences of the University “Magna Graecia” of Catanzaro [12]. Recruited subjects participated to a campaign for assessment of cardio-metabolic risk factors. Recruitment mechanisms include word-of-mouth, fliers, and newspaper advertisements. The inclusion criteria were: fasting plasma glucose <126 mg/dl and presence of one or more cardio-metabolic risk factors including, hypertension, dyslipidemia, and overweight/obesity. Subjects were excluded if they had chronic gastrointestinal diseases, chronic pancreatitis, history of any malignant disease, history of alcohol or drug abuse, positivity for antibodies to hepatitis C virus (HCV) or hepatitis B surface antigen (HBsAg), kidney or hepatic failure. After 12-h overnight fasting, subjects underwent anthropometrical evaluation and a venous blood sample was drawn for laboratory determinations. 

The study was approved by Institutional Ethics Committees of University "Magna Graecia" of Catanzaro and University of Rome-Tor Vergata. Written informed consent was obtained from each subject in accordance with principles of the Declaration of Helsinki.

### DNA analysis

DNA was isolated from whole blood using commercial DNA isolation kit (Promega, Madison, WI). Screening of rs35767 polymorphism was performed using a TaqMan allelic discrimination assay (Applied Biosystems, Foster City, CA). TaqMan genotyping reaction was amplified on a GeneAmp PCR system 2700 and fluorescence was detected using an ABI Prism 7000 sequence detector (Applied Biosystems, Foster City, CA). Genotyping quality was tested by including 3 HapMap samples in each 96-well plate. The agreement rate with the HapMap database genotypes was >99%. 

### Analytical determinations

Glucose concentrations were measured by enzymatic methods (Hoffman-La Roche, Basel, Switzerland). Serum insulin concentration was determined by a chemiluminescence-based assay (Immulite^®^, Siemens, Italy). Total serum IGF-1 concentrations were determined by chemiluminescent immunoassay (Nichols Advantage®, Nichols Institute Diagnostic, San Juan Capistrano, CA) for samples collected until 2008. Because the Nichols Advantage® system has since been discontinued and is no longer available, total IGF-1 was subsequently assayed by one-step sandwich chemiluminescence immunoassay (CLIA) after prior separation of IGF-I from binding proteins on the Liaison® autoanalyzer (DiaSorin, Saluggia, Italy). Intra-assay and inter-assay CV were 4.4 and 5.5%, respectively. 65 blood samples from study subjects of both sexes were used to compare the two assay methods (the Nichols Advantage® system vs. Liaison® DiaSorin). The correlation between the two methods was r=0.987, which yielded similar results for IGF-1 concentration.

### Calculations

Glucose disposal (M) was calculated as the mean rate of glucose infusion measured during the last 60 min of the clamp examination (steady-state) and is expressed as milligrams per minute per kilogram fat-free mass (M_FFM_) measured with the use of electrical bioimpedance. Insulin clearance was calculated by dividing the rate of insulin infusion by the mean steady-state plasma insulin concentration (SSPI) during the insulin infusion as previously described [13]. The homeostasis model assessment (HOMA) index was calculated as fasting insulin × fasting glucose/22.5 [14].

### Statistical analysis

The results for continuous variables are given as means ± SD. Categorical variables were compared by χ^2^-test. Differences of continuous variables between groups were tested after adjusting for age, gender, and BMI by ANCOVA (general linear model). The Hardy-Weinberg equilibrium between the genotypes was evaluated by χ^2^ test. Genotype distributions were in Hardy-Weinberg equilibrium (*P*>0.01). Power calculations were performed with Quanto version 1.2.4 (http://hydra.usc.edu/gxe; accessed 25 July 2011). The study had 81% power (for α=0.05) to detect a 17 ng/ml change in IGF-1 plasma levels per allele A in sample 1, and 80% power (for α=0.05) to detect a 10 ng/ml change in IGF-1 plasma levels per allele A in sample 2 according to a dominant model. The study had 82% power (for α=0.05) to detect a 1 mg x Kg^-1^ x min^-1^ change in insulin-stimulated glucose disposal per allele A in sample 1 according to a dominant model. Associations between the rs35767 polymorphism and continuous traits are presented as effect sizes (β and SE) per insulin–raising allele, estimated by multiple linear regression analysis adjusted for gender, age, and BMI. We report nominal *P* value <0.05 without adjustment for multiple testing given the high prior probabilities for association of the rs35767 polymorphism with the examined traits (the locus has already been associated with insulin levels, a proxy of insulin sensitivity, at genome-wide levels of statistical significance [*P*< 5 x 10^-8^]). All analyses were performed using the SPSS software program Version 16.0 for Windows.

## Results

Clinical characteristics of sample 1 and sample 2 according to the SNP rs35767 near *IGF1* are shown in Table 1. Because of the small number of AA individuals and the a priori hypothesis based on the dominant effect observed in previous studies [1], associations between the rs35767 polymorphism and continuous traits were analysed according to both a dominant and an additive genetic model. In sample 1, the rs35767 polymorphism did not show any significant association with age, gender, BMI, fasting plasma glucose, and insulin clearance, whereas it was significantly associated with fasting plasma insulin levels (Table 1). IGF-1 levels were significantly lower in carriers of the GG genotype as compared with subjects carrying the A allele (190±77 vs. 218±97 ng/ml, respectively; *P*=0.007 after adjusting for age, gender, and BMI). Insulin sensitivity assessed by euglycaemic-hyperinsulinemic clamp was significantly lower in carriers of the GG genotype as compared with subjects carrying the A allele (8.9±4.1 vs. 10.1±5.1 mg x Kg^-1^ free fat mass x min^-1^, respectively; *P*=0.03 after adjusting for age, gender, and BMI) (Table 1). To estimate the independent contribution of the rs35767 polymorphism to insulin sensitivity, we carried out a linear regression analysis in a model which also included gender, age, BMI, and fasting plasma glucose. Comparison of standardized coefficients allowed the determination of the relative strength of each trait’s association with insulin sensitivity (listed from strongest to weakest): BMI (β= -0.32, *P*<0.0001), male gender (β= -0.28, *P*<0.0001), and the rs35767 polymorphism (β=0.08, *P*=0.03). These factors explained 22% of the variance in insulin sensitivity. Interestingly, after including plasma IGF-1 concentrations in the regression model, the variables which remained independently associated with insulin sensitivity were BMI (β= -0.30, *P*<0.0001), and male gender (β= -0.29, *P*<0.0001), while the rs35767 polymorphism was excluded. 

**Table 1:** Clinical characteristics of 569 study subjects (sample 1) and of 852 study subjects (sample 2) according to the SNP rs35767 near IGF1.

	**Variables**	**GG**	**GA**	**AA**	***P***	***P (G/G vs. G/A+A/A)***	***β***	**SE**	***P***
**Sample 1**	Male/Female	184/229	56/79	10/11	0.77	0.63			
	Age (*yr*)	39±10	37±10	37±7	0.19 §	0.08 §			
	BMI (*kg/m* ^*2*^)	30.1±7.3	28.9±6.8	28.4±6.1	0.28 #	0.12 #			
	Fasting Glucose (*mg/dl*)	92±11	90±11	90±10	0.89*	0.64*			
	Fasting Insulin (*μU/ml*)	13±10	12±7	9±4	0.12*	0.05*	-0.08	1.02	0.05
	IGF-1 (*ng/ml*)	190±77	217±99	219±97	0.02*	0.007*	0.12	8.7	0.007
	Insulin-stimulated glucose disposal (*mg x Kg* ^*-1*^ * FFM x min* ^*-1*^)	8.9±4.1	10.0±5.3	11.0±3.6	0.05*	0.03*	0.08	0.37	0.03
	Insulin clearance (*ml/min x m* ^*2*^)	521±238	572±392	567±215	0.50*	0.24*			
**Sample 2**	Male/Female	255/307	116/136	18/27	0.75	0.84			
	Age (*yr*)	49±13	50±14	48±11	0.58§	0.38§			
	BMI (*kg/m* ^*2*^)	29.1±5.6	29.1±6.1	29.4±4.9	0.87#	0.84#			
	Fasting Glucose (*mg/dl*)	88±7	88±7	89±6	0.24*	0.64*			
	Fasting Insulin (*μU/ml*)	13±10	11±6	10±5	0.07*	0.03*	-0.07	0.63	0.03
	IGF-1 (*ng/ml*)	154±60	163±66	169±62	0.01*	0.004*	0.09	3.99	0.004
	HOMA index	2.8±2.2	2.5±1.4	2.3±1.1	0.07*	0.03*	-0.07	0.14	0.03

Data are means ± SD. Differences of continuous variables between groups were tested after adjusting for age, gender, and BMI by ANCOVA (general linear model). Categorical variables were compared by χ^2^ test. § *P* values refer to results after analyses with adjustment for gender. #*P* values refer to results after analyses with adjustment for age, and gender using a general linear model. *P values refer to results after analyses with adjustment for age, gender and BMI using a general linear model. Effect sizes (β and SE) per allele A and corresponding *P* values are shown.

In order to get further insights on the role of the rs35767 polymorphism on IGF-1 levels and insulin sensitivity, an additional sample of nondiabetic individuals was analysed. As shown in Table 1, in sample 2, the rs35767 polymorphism did not show any significant association with age, gender, BMI, and fasting plasma glucose, whereas it was significantly associated with fasting plasma insulin levels (Table 1). IGF-1 levels were significantly lower in carriers of the GG genotype as compared with subjects carrying the A allele (155±60 vs. 164±63 ng/ml, respectively; *P*=0.02 after adjusting for age, gender, and BMI). Insulin sensitivity, as estimated by the HOMA index, was significantly lower in carriers of the GG genotype as compared with subjects carrying the A allele (2.8±2.2 vs. 2.5±1.3, respectively; *P*=0.03 after adjusting for age, gender, and BMI) (Table 1).

## Discussion

In the present study we investigated the effect of the rs35767 polymorphism near *IGF1*, previously associated with fasting insulin levels, on circulating IGF-1 levels, and insulin sensitivity in 2 cohorts with over 1400 white European participants. We found that the rs35767 polymorphism was associated with circulating IGF-1 levels with carriers of the GG genotype exhibiting lower concentrations as compared with subjects carrying the A allele. In addition, we confirm that carriers of the GG genotype have lower insulin sensitivity as compared with subjects carrying the A allele using the gold-standard technique, the euglycaemic–hyperinsulinaemic clamp method rather than surrogate indexes based on glucose and insulin measured in the fasting state or during an oral glucose tolerance test [1,7]. In a linear regression analysis, the rs35767 polymorphism was significantly associated with insulin sensitivity independently of age, gender, BMI, and fasting glucose levels. Notably, inclusion of circulating IGF-1 levels in the regression model resulted in the exclusion of the rs35767 polymorphism from the variables explaining the variability in insulin sensitivity; this suggests that the polymorphism near *IGF1* was affecting insulin sensitivity by regulating plasma IGF-1 levels. 

IGF-1, which has significant amino acid sequence homology with insulin, enhances insulin sensitivity in both animal models and human subjects acting through IGF-1 receptors and/or hybrid insulin/IGF-1 receptors [3,15,16]. Additionally, low circulating IGF-1 levels might lead to up-regulation of IGF-1 receptors content resulting in increased formation of hybrid insulin/IGF-1 receptors, whose assembly is function of the relative number of insulin and IGF-1 receptors [17-19]. Consequently, increased formation of hybrid insulin/IGF-1 receptors that bind insulin with low affinity may contribute to impair insulin action by sequestering insulin receptors in a less responsive form [17-19]. 

Alternatively, low circulating IGF-I levels may provide insufficient negative feedback at the level of the hypothalamus and/or pituitary thus resulting in compensatory over-secretion of growth hormone (GH) and decreased insulin sensitivity [20]. Accordingly, it has been shown that blocking GH action in mice with liver-specific deletion of the *IGF1* gene by crossing them with mice expressing a GH antagonist transgene, which prevents GH activation of its receptor, resulted in improved insulin sensitivity and glucose homeostasis [20]. Thus, these results suggest that GH hypersecretion may be a major determinant of lower insulin sensitivity in carriers of the GG genotype characterized by low circulating IGF-1 levels.

It has been recently reported that another polymorphism near *IGF1* (rs35749) is associated with insulin clearance [8]. We found that insulin clearance did not differ between carriers of the GG genotype and subjects carrying the A allele of the rs35767 polymorphism, thus excluding the possibility that raised fasting insulin levels observed in carriers of the GG genotype was mediated by variation in insulin clearance. 

The strengths of the present study include the replication of the association between the rs35767 polymorphism and circulating IGF-1 levels in two independent cohorts, the use of the gold standard euglycemic hyperinsulinemic clamp for assessment of insulin sensitivity, the detailed measurements of insulin clearance, the exclusion of subjects with conditions that affect glucose metabolism, the strict quality control of data collection by a trained staff following a standardized protocol [10,11], and the centralized measurements of biochemical variables.

Nevertheless, some limitations should be acknowledged in the interpretation of our results. One limitation is that euglycemic hyperinsulinemic clamp studies were only performed once. Although such an approach is common in clinical research due to the fact that the clamp technique is invasive, expensive, time consuming and not suitable for large-scale studies, intra-individual variation in insulin sensitivity cannot be taken into account. Furthermore, circulating IGF-1 levels were measured once, a common limitation to large epidemiological studies, and may not reflect IGF-I local tissue concentrations, which were not assessed in this study. Moreover, multivariate analysis included all subjects, although there were minor differences in insulin sensitivity between males and females. However, gender differences were adjusted for in the multivariate models. Additionally, the factors that we considered together explained 22% of the overall variation in insulin sensitivity, suggesting the existence of other regulators of insulin sensitivity. Furthermore, although our findings are clinically and biologically plausible, causality cannot be inferred due to the cross-sectional design of the study, which precludes us from drawing conclusions about the causal relationships between the rs35767 polymorphism and insulin sensitivity. Finally, our findings may apply only to white Europeans, and should not be generalized to other ethnic populations. Therefore, the present data should be considered hypothesis generating and requiring confirmation by further prospective studies including individuals of other ethnic groups. Nevertheless, we consider our results important in attempting to understand the pathophysiological interaction between the rs35767 polymorphism near *IGF1* and insulin sensitivity.
